# Antimicrobial Activity of Eco-Friendly Fly-Ash-Based Geopolymer Mortar

**DOI:** 10.3390/ma18081735

**Published:** 2025-04-10

**Authors:** Zeynep Iyigundogdu, Hüsamettin Ürünveren, Ahmet Beycioğlu, Nabi Ibadov

**Affiliations:** 1 Department of Bioengineering, Adana Alparslan Türkeş Science and Technology University, 01250 Adana, Türkiye; 2 Department of Civil Engineering, Adana Alparslan Türkeş Science and Technology University, 01250 Adana, Türkiye; hurunveren@atu.edu.tr (H.Ü.); abeycioglu@atu.edu.tr (A.B.); 3 Faculty of Civil Engineering, Department of Production Engineering and Construction Management, Warsaw University of Technology, Al. Armii Ludowej 16, 00-637 Warszawa, Poland

**Keywords:** geopolymer, mechanical properties, antimicrobial activity, curing temperature

## Abstract

As cement production causes large amounts of CO_2_ emissions and is not sustainable, there is a growing worldwide interest in developing cleaner construction materials by reducing carbon emissions and reusing existing industrial waste. Also, antimicrobially active construction materials are gaining attention due to enhancing structural longevity. By preventing microbial growth, these materials help to improve indoor air quality and occupant health. Geopolymer mortars/concretes (GPM/GPC) with high mechanical, physical and durability properties are considered as an eco-friendly alternative to ordinary Portland cement (OPC) mortars/concretes. In this study, the composition, microstructural, mechanical and antimicrobial properties of geopolymers produced at different curing temperatures (60, 80, 100 and 120 °C) were investigated. Low-lime fly ash was used as binder and sodium silicate and sodium hydroxide were used as the alkaline solution in geopolymer production. Although X-ray fluorescence (XRF) results showed an increase in geopolymerization products with increasing temperature, SEM analysis showed that the crack formation that occurs in the microstructure of geopolymers cured above 100 °C leads to decreased mechanical properties. The strength and antimicrobial performance test results for geopolymer mortars showed that the optimum temperature was 100 °C, and the highest compressive strength (48.41 MPa) was reached at this temperature. A decrease in strength was observed due to cracks occurring in the microstructure at higher temperatures. The agar diffusion method was used to determine the antimicrobial activity of GPMs against four bacteria and one fungus species. The antimicrobial activity test results showed that the samples subjected to thermal curing at 100 °C formed the highest inhibition zones (38.94–49.24 mm). Furthermore, the alkalinity of the components/mixtures has a direct relationship with antimicrobial activity. As a result, GPMs with superior antimicrobial and mechanical properties can be considered as promising building materials, especially for construction applications where hygiene is a priority and for structures that are likely to be exposed to microbial corrosion.

## 1. Introduction

Concrete, one of the most widely used construction materials, has been investigated in depth in terms of microstructure, macro-properties, environmental effects and mechanical and durability performances. The concrete production process demands vast amounts of resources, leading to serious environmental concerns such as high energy consumption and substantial carbon emissions. Researchers have long pointed out that making cement is far from eco-friendly, largely due to its intense energy requirements [1,2]. For instance, producing one ton of cement releases nearly an equivalent amount of CO_2_ and consumes about 94.76 million joules of energy [3,4]. In fact, cement production accounts for roughly 5–8% of the total CO_2_ emissions globally, and it ranks among industries with the highest emissions per dollar of economic output, contributing significantly to global CO_2_ levels and air pollution through the release of harmful greenhouse gases [5,6,7,8,9].

Globally, around 2.8 billion tons of cement were produced in 2006, and projections suggest that this number will exceed 4 billion tons by the 2050s. Rapid urbanization, growing populations and expanding infrastructure needs are driving up the demand for cement, which in turn makes reducing its environmental impact even more challenging [8]. Considering OPC production-oriented environmental damage, developing alternative materials to replace OPC has great importance for researchers.

**Figure 1:**
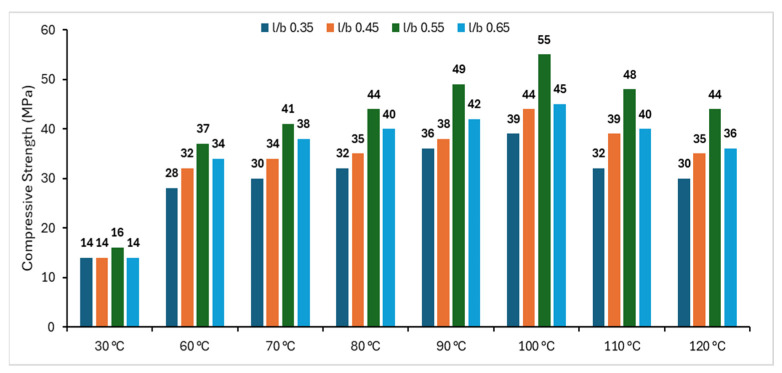
Effect of different oven curing temperatures (l/b: activator/binder) [22].

Geopolymer (GP) is a material that is offered as an alternative for OPC by Davidovits et al. due to its good fire resistance and good strength retention [8,9,10]. GP is a large complex produced by the covalent bonding of large oligomers via a geopolymerization reaction. In the geopolymerization reaction, silicate, alumina, carbonate or sulphate is activated by using strong alkaline solutions such as sodium hydroxide (NaOH), sodium silicate (Na_2_SiO_3_), etc., and the final structure of the GP is a combination of SiO_4_ and AlO_4_ tetrahedrons. GPs offer a number of advantages over conventional OPC, especially in terms of CO_2_ emission reductions [6]. GPs are envisioned as a good alternative to reduce the environmental hazards of the traditional cement industry and could decrease CO_2_ emissions by around 50–80% by 2050. Furthermore, industrial wastes such as fly ash (FA) or slag can be incorporated into the geopolymerization reaction as a binder material in the presence of an alkaline solution. This can be considered as upcycling of industrial wastes [11,12,13]. The mechanical performance of geopolymer-bound composites depends on the aluminosilicate source used in the mixtures, the type of alkali activator solution and the amount of mixture, while the curing temperature conditions are also an important parameter in determining the mechanical performance [3,14,15]. Low-calcium FA (Class F FA-based) geopolymer concrete requires slightly elevated temperatures (usually <100 °C) during the curing period [16]. When geopolymer mortars are subjected to thermal curing at temperatures of up to 100 °C, the physically bound water simply evaporates, while the trapped water inside starts to turn into vapor. When the temperature rises above 100 °C, the vapor pressure creates pressure in the material. However, when the mentioned temperature threshold of 100 °C is exceeded, even the chemically bound water in the structure starts to evaporate and the hydroxyl groups (OH) are removed through dehydration and dihydroxylation processes [17]. Using higher temperatures during curing (typically between 60 °C and 100 °C) can significantly improve the early compressive strength of FA-based geopolymer mortars because the elevated temperature accelerates the geopolymerization reaction [18]. The literature shows that the polymerization reaction, with increasing temperature, generally leads to the formation of structural cracks when it exceeds 100 °C [16,19,20,21]. Hemn Unis Ahmed et al. [22] graphically presented the temperature–compressive strength relationship in a comprehensive manner, taking into account the experimental work of Joseph and Mathew [19] (Figure 1).

As can be seen in Figure 1, the compressive strength increased parallel to the increase in temperature in all variable parameters up to 100 °C and decreased after 100 °C. Concrete degradation is typically assessed by evaluating its resistance to acidic chemicals, sulfate attack, high temperatures, chloride-induced corrosion, atmospheric exposure, carbonation, alkali–silica reactions and freeze–thaw cycles. For geopolymer concrete to serve as a viable alternative to OPC, it must demonstrate durability performance that is comparable to or superior to that of conventional concrete [23,24]. According to the literature, unlike OPC hydration products, geopolymer concrete (GPC) is chemically stable and does not undergo structural breakdown when exposed to high temperatures. Its absorption rate is significantly lower than that of conventional OPC concrete due to the amorphous particles formed during the geopolymerization process, which fill the pores, reducing porosity and increasing the microstructural density. Chloride resistance in GPC is directly related to its ability to bind chloride ions, preventing their penetration and diffusion. The selection of an appropriate alkali activator and optimized curing conditions can further enhance GPC’s durability by refining pore structure, increasing polymerization, and generating more N(C)–A–S–H gels that effectively adsorb free chloride ions, ultimately improving chloride resistance. Moreover, GPC demonstrates superior chemical stability compared to OPC concrete. Its geopolymerized bonding structure is more resistant to sulfuric acid degradation than the cementation bonding in OPC. GPC also exhibits greater sulfate resistance due to the stability of its cross-linked silicate gel, which remains intact in sulfate solutions. In contrast, the C–S–H gel in OPC concrete dissolves in sulfate solutions, leading to decalcification and the formation of gypsum or ettringite. Under freeze–thaw conditions, both the capillary absorption rate and saturation degree of GPC can increase. However, by developing a dense pore structure and enhancing the polymerization of the binder material, GPC’s resistance to freeze–thaw cycles can be significantly improved. The potential occurrence and severity of ASR in GPCs are suggested to be lower than in OPC concretes due to the formation of a dense bond around the aggregates following the initial reaction. Alkalis participating in the chemical reaction are absorbed by the amorphous component of fly ash, transforming into cementitious binders and zeolite crystals. Any excess alkalis then react with the aggregates, initiating ASR while the material remains in gel form. However, the resulting silica gel (alkali-aggregate product) does not appear to be expansive, likely due to the limited availability of calcium, which typically promotes ASR. Additionally, ASR reactions have been suggested to contribute to a stronger bond at the paste–aggregate interface, potentially enhancing the tensile strength of GPCs [25,26].

Materials that have the ability to inhibit or kill microorganisms such as bacteria, fungi, viruses, etc., can be defined as antimicrobial materials, and their importance is constantly increasing due to the growing public awareness of infectious diseases [27]. These materials play an important role in many industries such as health and medical applications, water treatment, food packaging, building materials, etc., due to their ability to inhibit the growth and spread of microorganisms [28]. Focusing on building materials, to date, there have been many attempts to develop antimicrobial building materials to contribute to a healthier and safer environment for occupants. Concrete is the most widely used building material. Despite the numerous advantages of concrete, the organic matter present in the concrete structure creates a very favorable environment for microbial growth. In addition, the capillary structure of concrete, which allows water to pass through the structure, acts as a transport channel for microorganisms throughout the entire building [6,29].

Antimicrobial properties are a very important issue in terms of both increasing the structural durability of concrete and protecting public health. In environments where hygiene is of high importance, such as sewage systems, marine facilities and healthcare facilities, microbial colonization can lead to biofilm formation on concrete surfaces and subsequent concrete deterioration due to microorganisms settling and spreading on these surfaces; this not only compromises structural integrity but also poses significant health risks. Furthermore, antimicrobial concrete has a wide range of application potential, from ensuring hygiene in hospitals and food processing facilities to extending the life of wastewater systems, thus contributing to sustainable construction practices and reducing maintenance costs [30,31,32]. For these reasons, there are many studies in the literature about the development of antimicrobial building materials. Iyigundogdu and Saribas [33] investigated the effect of boron addition into cement mortar on its mechanical strength and antimicrobial activity. They observed that the boron-added mortars demonstrated substantial antimicrobial properties against *Candida albicans* and *Aspergillus niger*; however, the strength of these mortars was decreased by between 7.41% and 31.5%. Slosarczyk et al. [34] presented a comprehensive study on the microbial growth and reproduction of building materials using metal and metal oxide nanoparticles, such as silver (Ag), titanium dioxide (TiO_2_), and zinc oxide (ZnO). They demonstrated that these nanoparticles penetrate the cell membranes of microorganisms and inactivate them. They found that materials containing ZnO and TiO_2_ exhibited antibacterial properties due to their photocatalytic effects. Rosendo et al. [35] examined the antimicrobial activity of ZnO/palygorskite-based building materials against Gram-negative and Gram-positive bacteria. They determined that, in addition to the photocatalytic effect of ZnO, palygorskite provided high resistance to bacteria due to its large surface area. Dyshlyuk et al. [36] examined the effects of ZnO, TiO_2_ and SiO_2_ nanoparticles on biological deterioration in building materials and showed that ZnO inhibited microorganisms such as *Bacillus subtilis* and *Aspergillus niger* more effectively than TiO_2_ and SiO_2_. Kong L et al. [37] reported that concrete activated using alkali activators can effectively resist microbial attack and could be applied in aggressive environments where conventional concrete often fails.

Building materials, especially those used in humid environments, are very susceptible to microbial colonization, and the microbial load in structures is associated with the deterioration of the structure. This study aims to develop more sustainable metal-free antimicrobial GP materials that can be used as a cement substitute. It is thought that structures built with antimicrobial GP will last longer without microbial corrosion and therefore have a lower landfill loading. Also, due to the lack of microbial load within the structure, structures built with antimicrobial GPs will have better indoor air quality and minimized repair costs due to microbial corrosion. For this purpose, in this study, a geopolymerization reaction was carried out at different curing temperatures using low-lime fly ash as a binder and a mixture of sodium silicate and sodium hydroxide as an alkaline solution. Characterization studies of the developed GPs were carried out and their mechanical strengths and antimicrobial activities were investigated.

## 2. Materials and Methods

### 2.1. Materials

Low-lime fly ash (FA) from İSKEN Thermal Power Plant (Yumurtalık, Adana, Türkiye) was used as the main powder material to produce geopolymer mortar as a binder. The alkali solution prepared in this study was a combination of sodium silicate (Na_2_SiO_3_) (SMS) and sodium hydroxide (NaOH). While the sodium hydroxide used in the study was of high purity, the sodium silicate also contained 10.40% Na_2_O and 22.05% SiO_2_. In addition to binder and activator components, CEN standard sand was used in mortar production. The properties of SMS and NaOH are given in Table 1, and the physical properties of FA are given in Table 2.

Four bacterial and one fungal species were used in antimicrobial activity studies. The microorganisms used in this study are given in Table 3. The microbial species were obtained from the American Type Culture Collection (ATCC, Manassas, VA, USA). The nutrient agar (NA) and potato dextrose agar (PDA) used in antimicrobial tests were purchased from Sigma-Aldrich (St. Louis, MO, USA).

Research manuscripts reporting large datasets that are deposited in a publicly available database should specify where the data have been deposited and provide the relevant accession numbers. If the accession numbers have not yet been obtained at the time of submission, please state that they will be provided during review. They must be provided prior to publication.

### 2.2. Sample Preparation

The MS (Silica Modulus) ratio is a very important factor in the workability and strength properties of GPMs [38]. The *MS* module is known simply as the silica module and is used in geopolymer mortar/concrete mixture calculations [39,40]. The following equation (Equation (1)) was used to calculate the amount of SMS and NaOH for the geopolymerization reaction of GPM mixtures [11,12,13].

(1) $$\begin{array}{l} MS=\frac{Total\;{SiO}_{2}}{Total\;{Na}_{2}O}\\ =\frac{\;{SiO}_{2}\;from\;Sodium\;Silicate}{{Na}_{2}Ofrom\;Sodium\;Silicate+{Na}_{2}Ofrom\;Sodium\;Hydroxite} \end{array}$$

Preliminary experimental studies conducted with different MS and Na_2_O (%) concentrations showed that preparing the mixtures with 1.4 MS and 10% Na_2_O content is appropriate to create workable mortars with adequate mechanical strength.

The alkaline solution, calculated according to the 1.4 MS module and 10% Na_2_O concentration, was prepared 24 h before the experiment and kept in the laboratory environment to cool down the solution for it to become ready for geopolymer production. After cooling, the alkaline solution was added to the fly ash and CEN standard sand mixture. Geopolymer mortar was prepared by mixing the alkaline solution and dry powder materials for 90 s, and cubic and cylindrical samples were cast with this mortar for strength and antimicrobial tests. The component amounts of GPMs used in the study are presented in Table 4.

**Figure 2:**
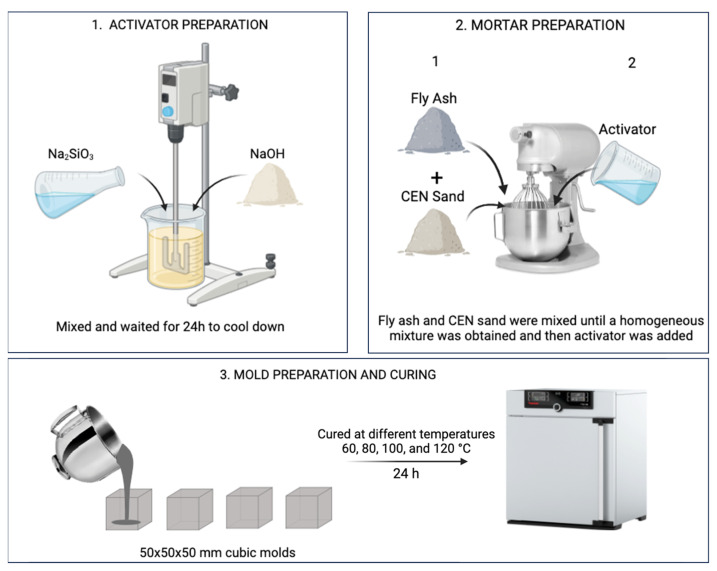
Schematic illustration of GPM production.

**Figure 3:**
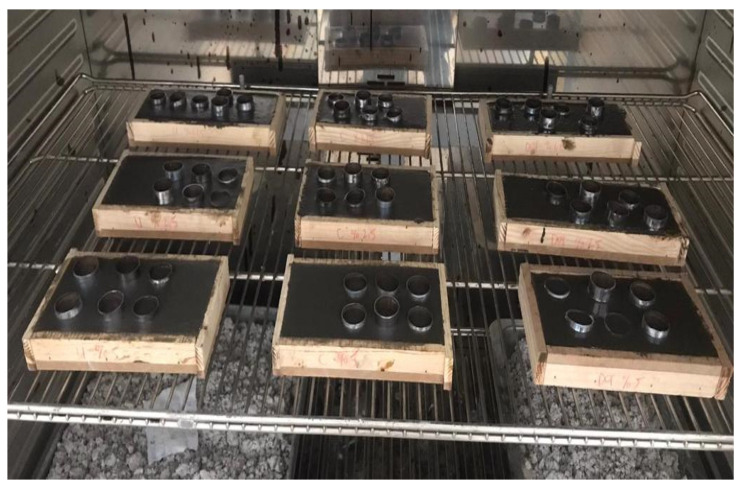
Cylindrical GPM samples for antimicrobial activity tests (oven curing stage).

Cubic GPM samples of 50 mm × 50 mm × 50 mm size for uniaxial compressive strength tests and cylindrical GPM samples with 20 mm diameter and 5 mm thickness for antimicrobial tests were prepared. All samples were cured in an oven at different temperatures (60, 80, 100 and 120 °C) for 24 h for the strength development of mortars. Curing temperature is an important parameter in the strength properties of geopolymer mortars. Since the strength development and antimicrobial performance properties of geopolymer mortars was to be investigated, curing at different temperature values was also added to the study as a parameter to evaluate these properties. After the curing process in the oven, the samples were removed from the molds and allowed to cool to room temperature before being subjected to the strength and antimicrobial tests. The GPM production procedure is schematized in Figure 2. The prepared cylindrical samples for antimicrobial activity tests are shown in Figure 3.

### 2.3. Characterization of FA and GPM

The microstructures of GPM samples cured at different temperatures were investigated using scanning electron microscopy (SEM). Before analysis, the specimens were sputter-coated with a gold–palladium alloy. The chemical compositions of FA and GPM samples were determined via the quantitative measurement method using an X-ray fluorescence (XRF) spectrometer (MiniPal 4, Malvern Panalytical, Almelo, The Netherlands).

### 2.4. Mechanical Test of GPMs

Compressive strength tests on the produced GPM samples were carried out using 50 mm × 50 mm × 50 mm cubic samples in accordance with TS EN 196-1 [41] and TS EN 12390-3 [42] and using a 0.6 MPa/s loading rate.

### 2.5. Antimicrobial Activity Studies

**Figure 4:**
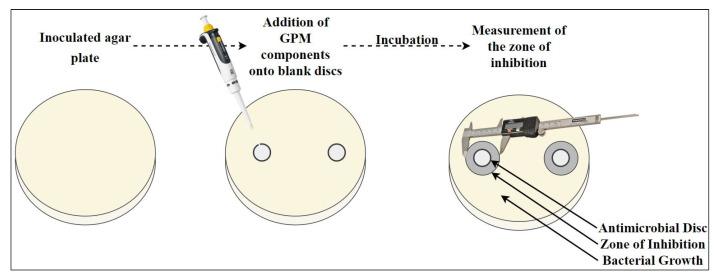
Schematic illustration of the disc diffusion method.

The agar diffusion method described by Li et al. was used against four bacteria and one fungus to determine the antimicrobial activity of GPMs cured at different temperatures (60, 80, 100 and 120 °C) [43]. First, each side of the GPM sample was UV sterilized for at least 1 h. Additionally, 0.5 McFarland bacterial suspensions were prepared in PBS and 2 McFarland fungi suspension was prepared in 0.5% Tween + PBS from fresh cultures. A volume of 100 μL of bacterial and fungal suspensions were spread on NA and PDA, respectively. GPM samples (approx. 20 mm diameter) were placed onto inoculated agar and placed into an incubator. Moreover, the components of the geopolymer and their mixtures, which are sodium SMS, SMS + NaOH mixture, FA and a mixture of all (SMS + NaOH + FA) were loaded onto a blank disc (approx. 20 μL for liquid and 10 µg for solid components) and placed onto inoculated mediums. The inoculated plates were incubated for 24 h for bacteria at (35 ± 1) °C and 72 h for fungus at (28 ± 1) °C. The antimicrobial activities were evaluated by measuring the clear zone around GPM samples. Results are expressed as the inhibition diameters (mm). A schematic illustration of the antimicrobial activity test is given in Figure 4.

## 3. Results and Discussion

### 3.1. XRF and XRD Analysis Results for FA and GPM

**Figure 5:**
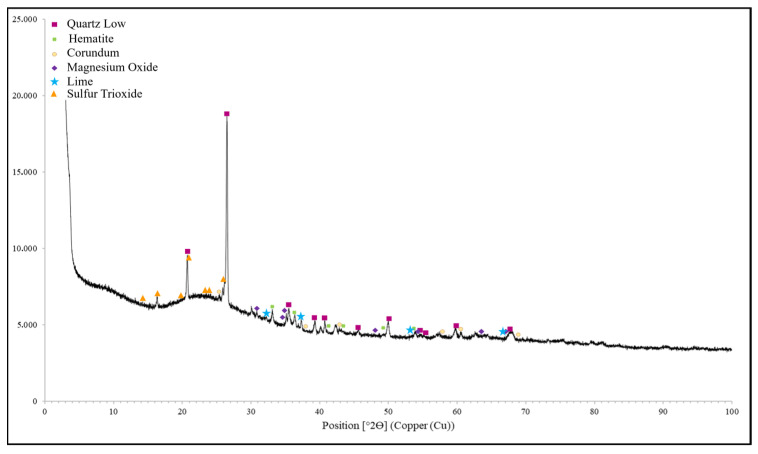
XRD analysis result for FA.

The XRF and XRD (X-Ray Diffractometer (Malvern Panalytical, Almelo, The Netherlands, Empyrean XRD)) results are given in Table 5 and Figure 5, respectively. As seen in Table 5, the sum of SiO_2_ + Al_2_O_3_ + Fe_2_O_3_ was 87.83% and the CaO ratio was 4.95% (<10%). According to ASTM C 618 [44], the FA used in this study is a low-lime FA and can be classified as F type. Quartz being found as a major component in XRD analyses confirms the XRF results.

Furthermore, XRF analysis was also carried out for all GPM samples cured at different temperatures to observe geopolymerization progress. According to the results of GPM samples, SiO_2_ increases while Al_2_O_3_ decreases with increasing temperature. This indicates that temperature plays an active role in the geopolymerization reaction.

### 3.2. Microstructure and Mechanical Properties of GPMs Cured at Different Temperatures

**Figure 6:**
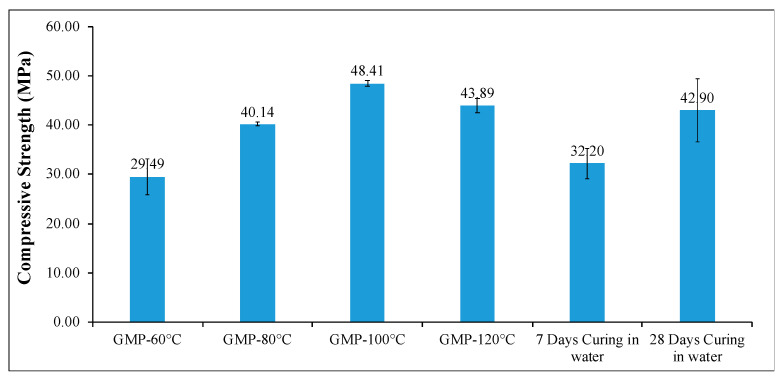
Compressive strength of GPMs and OPCMs.

The compressive strengths of all GPM samples cured at different curing temperatures are given in Figure 6. When the uniaxial compressive strength results are examined, it is observed that there is a significant increase in the strength values when the temperature is increased up to 100 °C, while there is a decrease in the strength values of geopolymer mortars cured at temperatures above 100 °C. The compressive strength value, which is 29.49 MPa at 60 °C, increased to 40.14 and 48.41 MPa at 80 and 100 °C curing temperatures, respectively. As can be understood from the SEM images, at temperatures above 100 °C, the strength value decreased to 43.18 MPa at 120 °C due to the inefficient formation of geopolymerization products and the increase in the number and size of the cracks observed in the microstructure.

**Figure 7:**
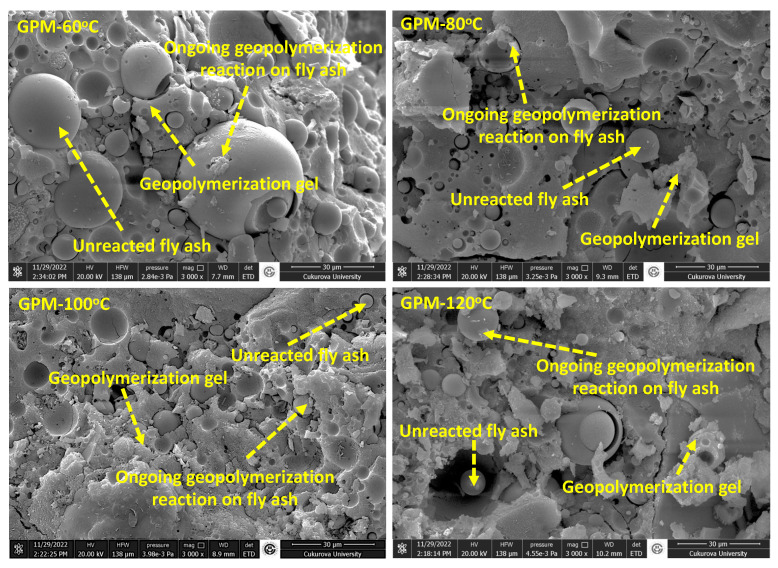
SEM images of GPM samples cured at different temperatures.

When the SEM images given in Figure 7 are examined, it is seen that more compact gel formations are formed and the amount of unreacted fly ash is relatively smaller in the GPM samples cured at increased temperatures. Although the geopolymerization reaction proceeds at higher temperatures, curing above 100 °C results in structural cracks within GPMs. These findings are consistent with previous studies in the literature. Palomo et al. reported that extended curing at higher temperatures has a negative effect on the compressive strength due to the collapse of the granular structure, resulting in desiccation and excessive shrinkage [45]. Joseph and Mathew studied the effect of the curing temperature of GPM and stated that compressive strength increases as the temperature is increased up to 100 °C; after 100 °C, increasing the curing temperature has a negative effect on mechanical performance [20]. According to Palomo et al., in alkaline activator solutions, the dissolution rate of aluminosilicate increases as the curing temperature is increased [45]. Luan et al. also recommend the necessary curing temperature to be at 100 °C or below [16]. Yang et al. stated that elevated curing temperatures increase kinetic energy, promote the degree of geopolymerization and produce a geopolymer with a stronger Al-Si-O network [46]. Kovalchuk et al. obtained similar results to Yang et al. and reported that elevated curing temperatures can help raise kinetic energy, thus facilitating the degree of polymerization, densifying the microstructure, and improving the mechanical properties of the geopolymer [47].

### 3.3. Antimicrobial Activity of GPM Samples

**Figure 8:**
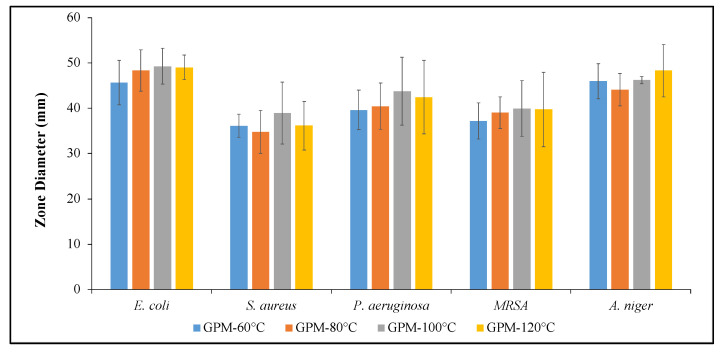
Inhibition zone diameters of GPMs cured at different temperatures.

**Figure 9:**
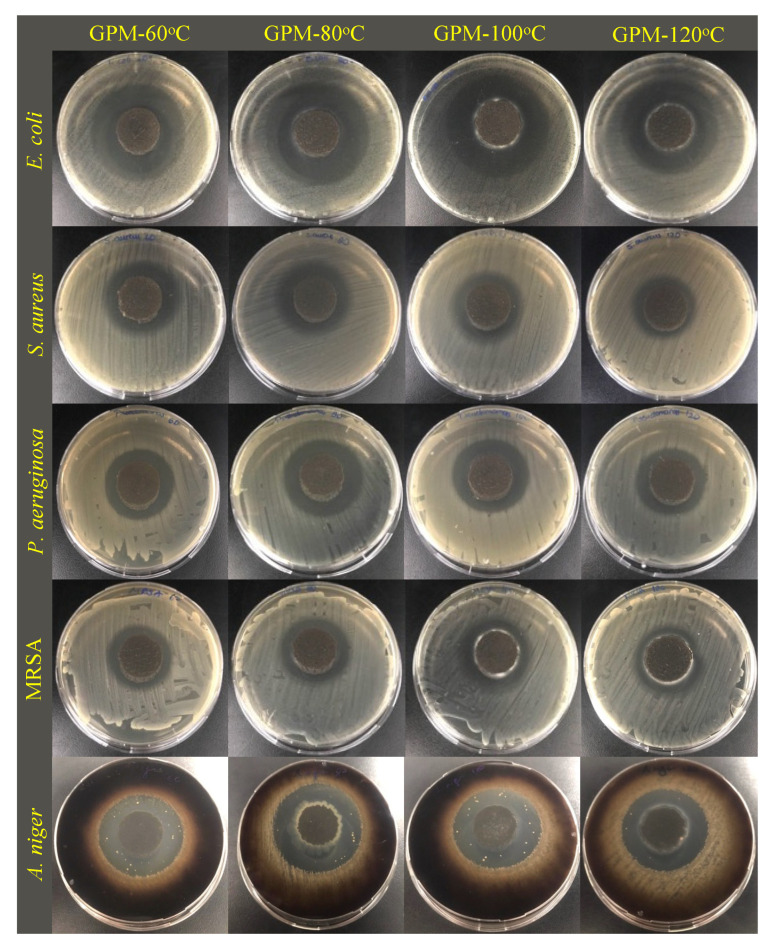
Agar diffusion test images of GPM cured at different temperatures.

According to the agar diffusion assay, all samples showed antibacterial and antifungal activity against all the tested microorganisms (Figure 8 and Figure 9), including an antibiotic-resistant bacterium (MRSA). Although there was no dramatic difference between the antimicrobial activity of bacterial strains, the highest inhibition zones were generally obtained against Gram-negative species (*E. coli* and *P. aeruginosa*).

In the literature, there are various compositions used to produce GPM [48,49,50,51,52,53,54]. Also, different additives such as copper, silver, zinc, titanium etc., were added to the geopolymer to produce antimicrobial activity [55,56,57,58]. In a study conducted by Ramadan et al., lead glass sludge including 10–35% lead oxide was used as a silica source and the antimicrobial activity of the GP produced was reported as antimicrobial due to the lead content in the structure [59]. In another study conducted by Tuntachon et al., TiO_2_ nanoparticles were added to produce antimicrobial activity to geopolymer paste [60]. In a Mexican patent, the antimicrobial activity of geopolymer was evaluated with different additives, and it was stated that plain geopolymer did not show antimicrobial activity [30]. However, the compositions and preparation methods of these geopolymers were different from the ingredients used in the present study.

**Figure 10:**
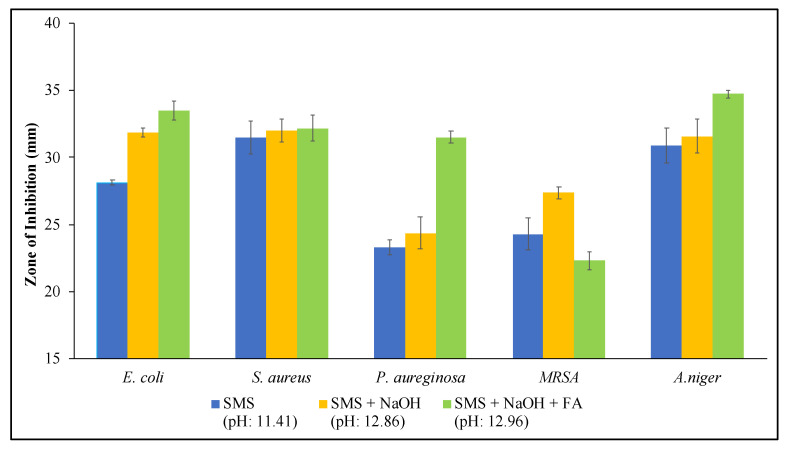
Agar diffusion test results of geopolymer components.

**Figure 11:**
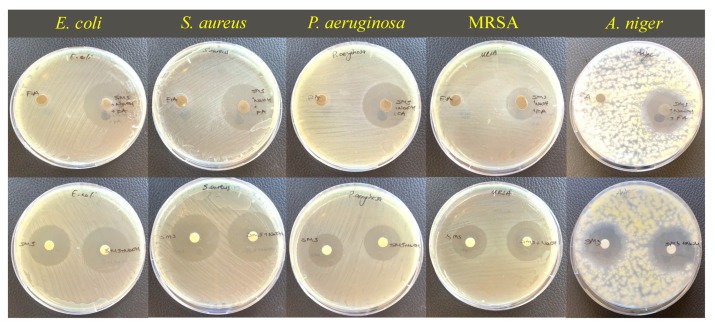
Agar diffusion test images of GPM components.

In order to determine the underlying reason for the antimicrobial behavior of the produced geopolymer mortars, the activities of all components used in the production of GPM were determined. The disc diffusion results obtained from geopolymer components and their mixtures are given in Figure 10 and Figure 11. According to the obtained results, SMS was the most active component, which has already been demonstrated in previous studies presented by Weber et al. and Basak et al. [61,62]. In addition, NaOH addition into SMS results in an increase in antimicrobial activity. It is known that microorganisms struggle to survive in highly alkaline environments due to the negative effects on their cellular components and metabolism (unless they are alkaliphiles). The reason for the increased antimicrobial activity is most likely related to the increased alkalinity of the mixture. The disc diffusion results also showed that, even if the FA itself did not show antimicrobial activity, zone diameters were increased for the SMS + NaOH + FA mixture. To understand the alkaline nature of solutions, the pH values of all materials and mixtures were measured. The pH results were recorded as 11.41, 12.86 and 12.95 for SMS, SMS + NaOH and SMS + NaOH + FA, respectively. The increase in the zone of inhibition diameters may be associated with the increased alkalinity of solutions. In alkaline environments, the cell membrane integrity and metabolic processes of microorganisms are disrupted, resulting in inhibition of microorganism growth or death [63,64].

Briefly, the antimicrobial activity of the produced GPM samples can be explained as, even if most of the alkali activator is involved in the geopolymerization reaction, a large amount of unreacted alkali solution still remains in the pores of the GPM [37]. The porous structure of geopolymers can leach unreacted alkaline solutions into the environment and these substances can disrupt the cellular functions of microorganisms and prevent microbial proliferation. Since the amount of leached solution will vary according to the polymerization and pore structure, the antimicrobial activity of GPM varies depending on these parameters.

## 4. Conclusions

In this study, the compositional, microstructural, mechanical and antimicrobial properties of low-lime-fly-ash-based geopolymer mortars were investigated. Four different curing temperatures (60, 80, 100 and 120 °C) were used to produce the mortars, and all properties of the mortars were determined for each curing temperature. Based on the experimental results obtained from this study, the following conclusions can be drawn.

- XRF analysis showed that SiO_2_ increased and Al_2_O_3_ decreased with increasing temperature. This means that temperature plays an active role in the geopolymerization reaction.
- There was a significant increase in the compressive strength values as the temperature was increased up to 100 °C, while the compressive strength values were reduced for samples cured at temperatures above 100 °C.
- The microstructural images obtained from SEM showed that more compact gel formations occurred in GPM samples produced at higher temperatures and the amount of unreacted fly ash was relatively smaller with respect to lower temperatures. Although geopolymerization was directly related to curing temperature, some structural cracks were observed in mortars cured at 120 °C. This implies that the high temperature damages the internal structure of the GPMs. The change in compressive strength results also support this result.
- Agar diffusion assays showed that all mixtures had antibacterial and antifungal activity against all tested microorganisms. The highest zone of inhibition against all tested microorganisms was obtained from the mixtures cured at 100 °C. The increase in inhibition zone diameters can be attributed to the increasing alkalinity of SMS, SMS + NaOH and SMS + NaOH + FA.
- This study emphasizes that the antimicrobial nature of geopolymer is directly related to the geopolymer components. There are many studies in the literature discussing the activity of geopolymers with various antimicrobial additives. However, this study shows, for the first time, that the antimicrobial activity of the geopolymer, of which components exhibit antimicrobial activity, continues due to the compounds remaining in the pores after polymerization reactions.

In this study, where the antimicrobial properties of geopolymer mortars were evaluated, it was found that geopolymer mortars, in addition to their high mechanical strength properties, have significant antimicrobial properties that make a significant contribution to the use of sustainable construction materials. Geopolymer mortars and concretes with antimicrobial properties have a wide range of potential uses, especially in civil engineering structures where hygiene is a priority. These materials, which are produced with lower carbon emissions compared to conventional cement composites, have become an important alternative in terms of providing a building stock that is both environmentally friendly, healthy and has a long service life with reduced maintenance costs.

## Figures and Tables

**Table 1:** Chemical properties of the activator’s components.

Sodium Silicate		Sodium Hydroxide	
Analysis	Specification	Analysis	Specification
Chemical formula	Na_2_SiO_3_	Degree	Extra Pure
Sodium oxide	10.40	Chemical formula	NaOH
Silicon oxide	22.05	Molar mass	40.00 g/mol
Density (20 °C)	1.439	Degree of purity	≥98.0%

**Table 2:** Physical properties of FA [12].

Feature	Density (kg/m^3^)	Specific Surface (cm^2^/g)	Activity Index (28 D (%))	Fineness (>45 μm)	Loss of Ignition (%)
**Value**	2295	4252	79.4	15.67	1.32

**Table 3:** Microbial species used in antimicrobial studies.

Bacteria
*Escherichia coli* (ATCC 8739)
*Pseudomonas aeruginosa* (ATCC 27853) *Staphylococcus aureus* (ATCC 6538)
Methicillin-resistant *Staphylococcus aureus* (MRSA) (ATCC 33592)
**Fungus**
*Aspergillus niger* (ATCC16404)

**Table 4:** The mixing ratios of the mortars according to the MS module.

FA (kg/m^3^)	Activator (kg/m^3^)		CEN (kg/m^3^)
**484.68**	Na_2_SiO_3_	NaOH	1454.037
	307.73	21.24	
	Total 328.976		

**Table 5:** Chemical properties of FA and GPM samples.

Sample Name	Component (%)							
	Al_2_O_3_	SiO_2_	SO_3_	K_2_O	CaO	TiO_2_	Fe_2_O_3_	Other
**FA**	21.00	53.00	1.50	2.70	4.95	1.31	13.83	1.71
**GMP-60 °C**	12	67	1.2	4.40	4.06	0.94	9.38	1.02
**GMP-80 °C**	13	67	1.0	4.59	3.80	0.88	8.79	0.94
**GMP-100 °C**	13	68	1.0	4.56	3.51	0.82	8.03	1.08
**GMP-120 °C**	12	71	0.7	4.36	3.09	0.75	6.97	1.13

## Data Availability

The original contributions presented in this study are included in the article. Further inquiries can be directed to the corresponding author.
